# LONELINESS TRANSITIONS AMONG MINORITY ETHNIC AND LGB POPULATIONS IN THE UK

**DOI:** 10.1093/geroni/igae098.0223

**Published:** 2024-12-31

**Authors:** Christina Victor, Isla Rippon

**Affiliations:** Brunel University London, London, England, United Kingdom; Brunel University London, London, England, United Kingdom

## Abstract

Loneliness has been identified as a major public health problem. Although there is a substantial body of research about loneliness in older adults in the UK, there is a significant evidence gap reporting experiences of loneliness among older people from ethnic minorities and those who identify as lesbian, gay, or bisexual (LGB). We focus upon the experiences of loneliness for adults aged 50+, from LGB and minority ethnic communities. Using waves 9-12 of the annual UK Household Longitudinal Study (UKHLS/Understanding Society) we measured loneliness using the three-item UCLA scale with a score of 6+ out of 9 defining loneliness. 7,646 respondents completed the loneliness measure at each wave. 1.7% of participants identified as LGB and 4.3% as Asian, 2.5% as black and 1.4% as mixed /other ethnicity. We grouped respondents into three categories: (a) consistently lonely; (b) consistently not lonely and (c) fluctuating loneliness. A higher proportion of LGB respondents were consistently lonely (16.4% vs 9.2%), compared to heterosexual respondents. Respondents from black (14.1%), Asian (11.2%) and other ethnic minorities (17.5%) were more likely to be consistently lonely in comparison to white respondents (8.9%). Shortcomings in data available on these groups of interest limit our analytical power to examine the importance of micro, meso and macro-level risk factors. Preliminary findings suggest that socio-demographic predictors for fluctuating or persistent loneliness differ in our groups of interest in comparison with white or heterosexual respondents. The higher level of persistent loneliness in the two groups has potential implications for their health and wellbeing.

